# Changes in saccadic eye movement and smooth pursuit gain in patients with acquired comitant esotropia after strabismus surgery

**DOI:** 10.16910/jemr.16.4.3

**Published:** 2023-09-22

**Authors:** Miharu Mihara, Atsushi Hayashi, Ken Kakeue, Ryoi Tamura

**Affiliations:** University of Toyama, Toyama, Japan

**Keywords:** Esotropia, eye tracking, saccade, smooth pursuit

## Abstract

This study investigates the change in horizontal saccadic eye movement and smooth pursuit in patients with acquired comitant esotropia (ACE), before and after strabismus surgery. The horizontal saccades and pursuit in 11 patients with ACE were recorded using a video eye-tracker under binocular viewing before and after strabismus surgery. Participants were instructed to fixate on the new target as rapidly as possible when it randomly appeared at either 18.3° rightward or 18.3° leftward. For smooth pursuit, participants were asked to track, as accurately as possible, a step-ramp target moving at ±6.1°/s. The asymmetry of adduction-abduction and the binocular coordination in gains of saccade and pursuit were compared between the pre- and post-surgical data. The asymmetry of adduction-abduction saccade gain in each eye after surgery tended to be smaller than that before surgery. The binocular coordination of saccade showed significant improvement after surgery in only the non-dominant eye direction. Adduction-abduction asymmetry in the smooth pursuit gain in each eye after surgery tended to be smaller than before surgery. After surgery, the binocular coordination of pursuit was improved significantly in both directions. In patients with ACE, binocular coordination of saccade and smooth pursuit was poor. Binocular coordination of saccade and pursuit seems to be improved due to the improvement in ocular deviation angle and binocular visual function after surgery.

## Introduction

It is known that even young infants can produce saccadic eye movements (10; 1). Though the gain and latency of saccades in infancy are immature, the saccade velocity is at the adult level (4). Voluntary saccades gain has also been reported to be not significantly different between the adult group and the group of children aged 4.5 to 12 years (24). The velocity and accuracy of saccades approaches adult values at an early age and stays stable after that (19). In contrast, smooth pursuit is immature at birth and develops well during infancy. However, smooth pursuit develops more slowly than saccades (13; 19); in particular, the gain is immature even in childhood.

There are several reports on the relationship between the development of eye movement systems and constant strabismus, such as infantile esotropia. Early-onset esotropia shows a greater asymmetry of pursuit gain (between temporal-to-nasal and nasal-to-temporal movement) than does late-onset esotropia (20; 17). A study for smooth pursuit in patients with horizontal strabismus concluded that binocular visual function is important in binocular coordination in pursuit (8). Although the details of voluntary saccades in esotropia are unknown, Bucci et al. investigated saccades in a group of patients with horizontal strabismus and found that binocular conjugacy and gain of the saccades were reduced (2) Furthermore, they reported that strabismus surgery improved saccades gain and binocular coordination (3)

However, it is not known in detail how ocular misalignment affects saccades and pursuit in patients with acquired comitant esotropia (ACE) presenting after childhood, as these patients have a developed ocular motor system. It is possible that, unlike early-onset esotropia, the saccades and smooth pursuit in patients with childhood or later esotropia onset change slightly or remain normal. However, this is not proven. Analysis of eye movements in childhood-onset ACE may provide a clue to the cause of onset. Moreover, it is unclear whether, if any abnormalities of the saccades and pursuit are found in patients with ACE in childhood, these abnormalities can be improved after correcting the ocular alignment. Therefore, in this study, we recorded horizontal saccades and pursuit in patients with childhood-onset ACE, characterized them, and examined their changes after strabismus surgery.

## Methods

### Participants

This study was approved by the Institutional Review Board of the University of Toyama (approval #27-159) and conformed to all local laws and the principles of the Declaration of Helsinki. Written informed consent was obtained from all participants after the experimental procedure was fully explained. In addition, for patients under 20 years of age, written informed consent was obtained from a guardian.

Patients with ACE scheduled to undergo strabismus surgery and individuals without strabismus participated in this study. Participants in this study were required to be older than 5 years of age. All patients with esotropia had no history of infantile esotropia or accommodative esotropia. All patients underwent surgery (unilateral recession of the medial rectus muscle with or without resection of the lateral rectus muscle in the non-dominant eye). All surgical procedures were performed by the same surgeon (MM). The deviation angles at near (30 cm) and far (5 m) were measured using the prism and alternate cover test. Stereopsis was evaluated using the Stereo Fly Test (Stereo optical CO., Inc, Chicago). Patients under the age of 35 were measured for cycloplegic refraction, and refraction was corrected as needed. The inclusion criterion was an esodeviation angle of ≥10 prism diopters (PD) at near and at far using the prism and alternate cover test, not including the A or V pattern strabismus. The dominant eye was determined using the Hole-in-Card test (18) All participants could clearly observe a visual target 40 cm away with either the naked eye or using soft contact lenses (corrected visual acuity 1.0 or greater). Patients who had other eye diseases, a neurologic or systemic disease related to strabismus, or eye movement disorder were excluded. All patients had normal magnetic resonance imaging (MRI) of the brain and orbit. All patients were examined before and three months after surgery.

Eleven patients with ACE (mean age: 27.4 ± 15.8 years, age range: 11–63 years, 5 females, Table 1) and 13 individuals without strabismus (mean age: 27.3 ± 14.5 years, range: 8–56 years, 6 females) volunteered to participate in this study. A 63-year-old male patient, who suffered from diplopia caused by esotropia for 40 years, was identified as not having sagging eye syndrome in an orbital MRI. Based on medical interviews and their photographs as infants, we judged that all patients became aware of diplopia and esotropia after 10 years of age.

### Materials & Procedure

The participants were seated in front of a table with their heads stabilized using a head positioner and were examined under binocular viewing. A computer monitor (Diamondcrysta^®^, RDT222WM-S; Mitsubishi, Tokyo) displayed the target located at 40 cm from the participant’s eyes. The monitor resolution was 1,680 × 1,050 pixels, and the refresh rate was 60 Hz. Each trial started with central fixation.

### Saccadic eye movement

**Figure 1.**
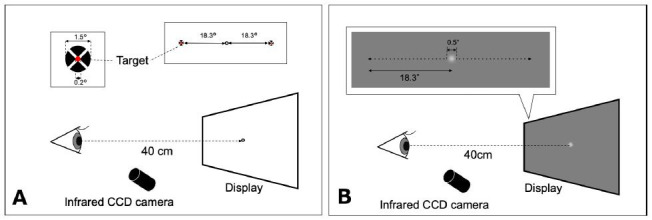
Schematic representations of the saccade (A) and smooth pursuit (B) tasks. The fixation target was displayed on a computer monitor at a distance of 40 cm. A: The target for saccadic eye movement appeared at either 18.3° rightward or leftward. B: The motion target of smooth pursuit moved from the center to either 18.3° leftward or 18.3° rightward. CCD: charge-coupled device.

The target of the saccade task consisted of a red inner circle (0.2° in diameter) superimposed at the center of a black circle (1.5° in diameter) and a white cross through the middle, on a white background (135 cd/m^2^) in accordance with Thaler et al. (22). The participants were instructed to fixate their gaze on an open black circle target (1.5° in diameter) located at the center at the beginning of each trial, and to fixate on the new target as rapidly as possible when it appeared. Finally, they returned their gaze to the center target when the latter target disappeared. The target of the saccade task randomly appeared at either 18.3° rightward or leftward of the center (Figure 1A). Each participant performed five trials in each direction. Data on eye movements that were in the wrong direction or contaminated with blinks were discarded. For each participant, the gain for adduction and abduction of each eye were calculated (mean of five trials).

### Smooth pursuit

The visual motion target for smooth pursuit testing was a white Gaussian spot with a diameter of 0.5° and luminance of 117 cd/m^2^ presented on a uniform gray background (70 cd/m^2^) in accordance with Ke et al. (6). Participants were asked to track a step-ramp target moving at ±6.1°/s horizontally as accurately as possible (Figure 1B). The step-ramp method was used to prevent early saccades in pursuit in accordance with the studies by Rashbass (14) and Sakuma et al. (15). The step amplitude was 0.85°. The target was initially displaced in the opposite direction of the target’s velocity step before moving back across the fovea ramp. The target moved from the center to either 18.3° leftward or 18.3° rightward. The direction was randomized across trials. Each participant performed five trials in each direction. Pursuit trials that included blinks, saccades (with the exclusion of catch-up saccades), or incorrect responses were excluded. A limited number of trials were performed due to patients being examined during consultation hours and the young age of some participants.

### Eye movement recording

The horizontal (X) and vertical (Y) positions of each eye were recorded using the ViewPoint EyeTracker^®^ system (Arrington Research, Scottsdale, AZ) at the sampling rate of 350 Hz and the spatial resolution of 0.15°. This system consisted of two infrared cameras mounted on the head positioner, which sent images of the eye to a computer via a USB cable. The eye tracker recorded the participants’ eye positions using the dark-pupil technique. The data were stored on a hard disk for off-line data analysis. Prior to testing, a 9-point grid (3 × 3 matrix) calibration and a subsequent validation procedure were performed for each participant using the software supplied by the eye tracker manufacturer.

### Data analysis and statistics

The saccade gain was the ratio of the amplitude of the saccade over the target amplitude. In this study, only primary saccades (not corrective saccades) were analyzed. The onset of a primary saccade was defined as the time when the eye velocity exceeded 5% of the saccadic peak velocity; the offset was set to the moment the eye velocity dropped below 10°/s, as is the previously reported standard (3; 2). The smooth pursuit gain was the ratio of the mean of eye movement velocity over the target velocity for each individual segment of smooth tracking, excluding catch-up saccade.

Means of the differences in the gain between the adduction and the abduction (Diff_Add-Abd_ gain; i.e., asymmetry of adduction-abduction) in each eye for saccade and smooth pursuit were calculated in each participant. Means of the differences in the gain between the dominant eye and the non-dominant eye (Diff_DE-NDE_ gain, e.g., adduction of dominant eye versus abduction of the non-dominant eye and abduction of dominant eye versus adduction of the non-dominant eye) for saccade and pursuit, i.e., binocular coordination, were calculated for each participant (across five trials in each trial type: movement direction to dominant eye side and to non-dominant eye side). The Wilcoxon signed-rank test was used to compare the Diff_Add-Abd_ and the Diff_DE-NDE_ gains between pre- and post-surgery. The Mann–Whitney U test was used to compare the Diff_Add-Abd_ and Diff_DE-NDE_ gains between the improvement and the unchanged groups in stereopsis post-surgery. The statistical analyses were performed using JMP Pro (version 14.2.0, SAS Institute, Cary, NC), and the significance level was set at P < 0.05.

## Results

### Changes in ocular deviation and sensory outcome after surgery

The mean presurgical esodeviation angles at near and far were 33.7 ± 14.8 PD (range: 14–56 PD) and 35.5 ± 10.4 PD (range 14–50 PD), respectively (Table 1). Some patients had esophoria during near vision and esotropia at a distance. The ocular deviations at near and far were 7.1 ± 4.6 PD (range: 0–14 PD) and 5.5 ± 4.4 PD (range: 0–12 PD), respectively, 3 months post-surgery (Table 1). The stereopsis at near pre-surgery in the patients varied from 50 to nil (more than 3000 seconds of arc) by Stereo Fly Test. Post-surgery, the stereopsis of all patients improved (more than two lines) or was equal (within one line) to that before surgery (Table 1).

**Table 1.** Summary of patients with acquired comitant esotropia.

No.	Age(y)/Sex	surgery	Pre-surgery			Post-surgery		
			Devistion Near(PD)	Deviation Far(PD)	Stereopsis (secarc)	Deviation Near(PD)	Deviation Far(PD)	Stereopsis (secarc)
1	35/M	R)MR5.5 LR6.0	35	40	100	8	8	50
2	15/M	L) MR6.0 LR6.0	55	50	>3000	4	4	80
3	16/M	L) MR4.0 LR4.0	20	30	>3000	4	0	40
4	35/F	R) MR5.0 LR5.5	40	35	100	14	4	100
5	23/F	L) MR6.0 LR6.0	41	46	800	12	8	800
6	29/F	L) MR6.5 LR6.5	56	40	100	8	10	60
7	63/M	R)MR7.0	20	30	60	2	0	80
8	11/F	R)MR4.5 LR5.5	20	35	400	10	12	400
9	17/M	L)MR4.5 LR4.5	25	25	50	0	0	50
10	14/M	R)MR5.0 LR6.0	45	45	400	12	10	400
11	43/F	R) MR6.5	14	14	100	4	4	40
mean	27.4		33.7	35.5		7.1	5.5	

M: male, F: female, R: right eye, L: left eye, MR: medial rectus muscle, LR: lateral rectus muscle, PD: prism diopter

### Changes in saccadic eye movement

**Figure 2.**
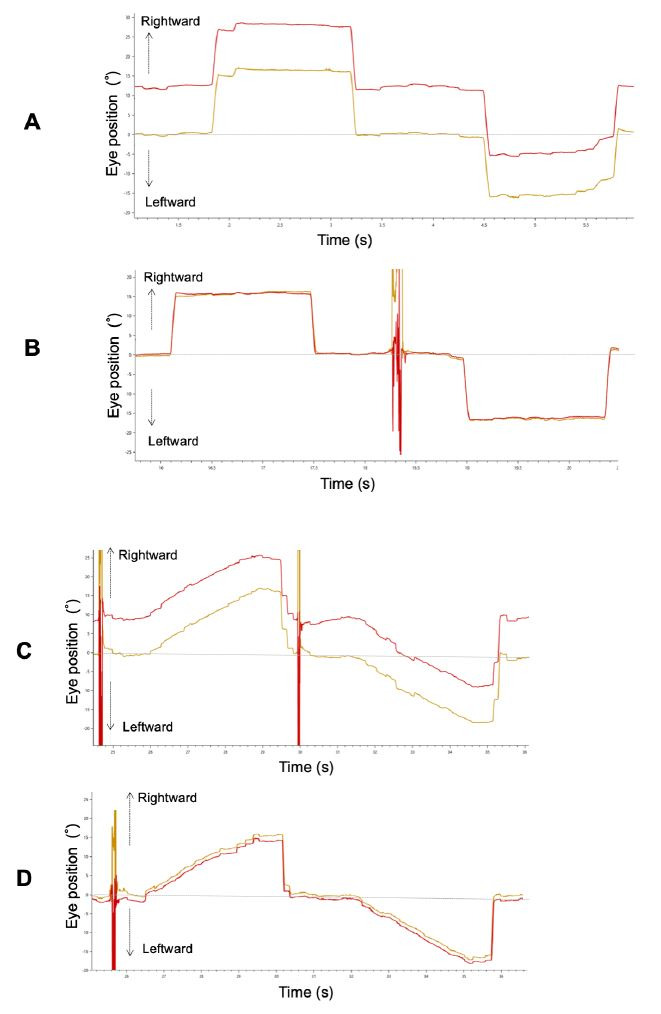
A typical example of a horizontal saccade (A: pre-surgery, B: post-surgery) and smooth pursuit (C: pre-surgery, D: post-surgery) waveform in Patient 3. Red and yellow lines indicate the left and right eye, respectively.

Figures 2A and B show binocular recordings of typical sequences of horizontal saccades for Patient 3 with ACE before and after surgery. The shape of the primary saccade was smooth for all participants. Before surgery, he fixed his right eye on the target, and his left eye deviated inward.

### Gain

**Figure 3.**
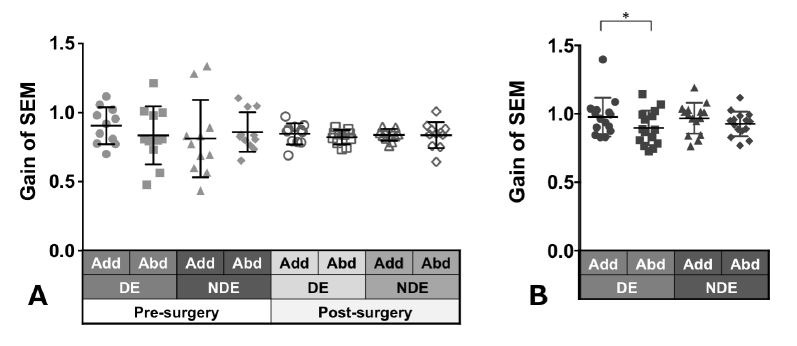
Comparison of pre- (solid gray symbols) and post-surgery (open gray symbols) saccade gain in patients with acquired comitant esotropia (A). Saccade gain in normal participants (B). Lines indicate mean with standard deviation. The asterisk (*) indicates *P* < 0.05. Add: adduction, Abd: abduction, DE: dominant eye, NDE: non-dominant eye, SEM: saccadic eye movement.

Patients with ACE: Figure 3A shows the gain of saccades in each eye and in each direction before and after surgery in patients with ACE. These values did not show a significant change between pre- and post-surgery, although variances decreased clearly in post-surgery. The decrease in adduction gain of the dominant eye post-surgery was the largest.

Normal individuals: Figure 3B shows the gain of saccades in each eye and in each direction in normal individuals and showed a tendency towards adduction dominancy, that is, gain of adduction larger than that of abduction (dominant eye: P = 0.04, non-dominant eye: P = 0.2).

### Asymmetry

**Figure 4.**
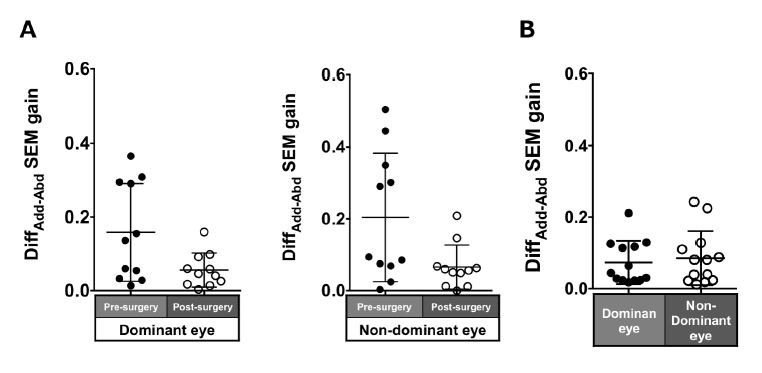
Comparison of pre- (solid black symbols) and post-surgery (open black symbols) asymmetry of adduction-abduction of both eyes in saccade gain in patients with acquired comitant esotropia (A). Asymmetry of adduction-abduction of both eyes in saccade gain in normal participants (B). Lines indicate mean with standard deviation. Diff_Add−Abd_: Difference between adduction gain and abduction gain, SEM: saccadic eye movement.

Patients with ACE: The Diff_ADD-ABD_ gain post-surgery in saccades in each eye was smaller compared to pre-surgery, although the difference was not significant (dominant eye: 0.16 ± 0.13 to 0.06 ± 0.05, P = 0.17; non-dominant eye: 0.2 ± 0.18 to 0.07 ± 0.06, P = 0.07; Figure 4A).

Normal individuals: The Diff_ADD-ABD_ gain in the normal individuals was smaller than the patients with ACE (dominant eye: 0.07 ± 0.06; non-dominant eye: 0.09 ± 0.08; Figure 4B) and showed a tendency toward adduction dominancy.

### Binocular coordination

**Figure 5.**
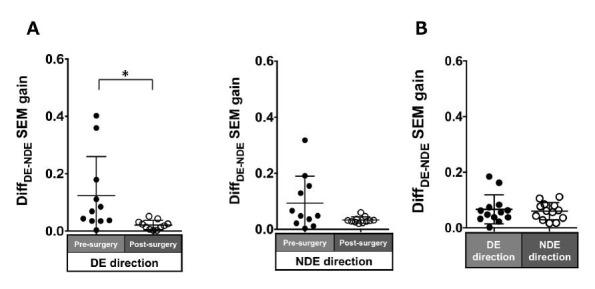
Comparison of pre- (solid black symbols) and post-surgery (open black symbols) binocular coordination of both eyes in saccade gain in patients with acquired comitant esotropia (A). Binocular coordination of both eyes in saccade gain in normal participants (B). Lines indicate mean with standard deviation. The asterisk (*) indicates P < 0.05. Diff_DE−NDE_: Difference between dominant eye gain and non-dominant eye gain, DE: dominant eye, NDE: non-dominant eye, SEM: saccadic eye movement.

Patients with ACE: In the pre-surgical data, Diff_DE-NDE_ gain in the dominant and non-dominant eye directions were 0.12 ± 0.14 and 0.09 ± 0.1, respectively. These values improved post-surgery but were significant in only the dominant eye direction (dominant eye direction: 0.02 ± 0.02, P = 0.007; non-dominant eye direction: 0.03 ± 0.01, P = 0.1; Figure 5A).

Normal individuals: The Diff_DE-NDE_ gain was small (dominant eye direction: 0.07 ± 0.05; non-dominant eye: 0.06 ± 0.03; Figure 5B).

### Changes in smooth pursuit

Figure 2C and D show a trajectory of both eye positions for Patient 3 with ACE before and after strabismus surgery. Before surgery, there was a difference in pursuit amplitude between both his eyes because he fixed her right eye on the target, and his left eye deviated inward as in the saccade. Because he maintained orthophoria during smooth pursuit after surgery, binocular coordination of smooth pursuit was improved.

### Gain

**Figure 6.**
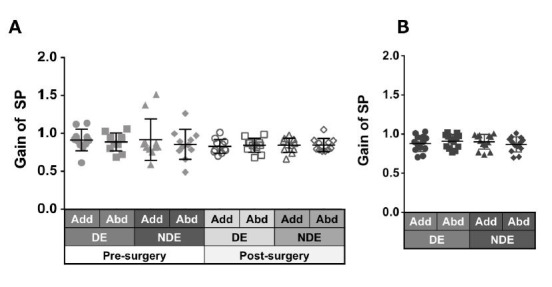
Comparison of pre- (solid gray symbols) and post-surgery (open gray symbols) smooth pursuit gain in patients with acquired comitant esotropia (A). Pursuit gain in normal participants (B). Lines indicate mean with standard deviation. Add: adduction, Abd: abduction, DE: dominant eye, NDE: non-dominant eye, SP: smooth pursuit.

Patients with ACE: Figure 6A shows the smooth pursuit gain in each eye and in each direction pre- and post-surgery. The pursuit gains, except in abduction of the non-dominant eye, decreased after surgery; however, the difference was not significant.

Normal individuals: Figure 6B shows the gain of pursuit in each eye and in each direction. Normal individuals showed gains of almost the same value.

### Asymmetry

**Figure 7.**
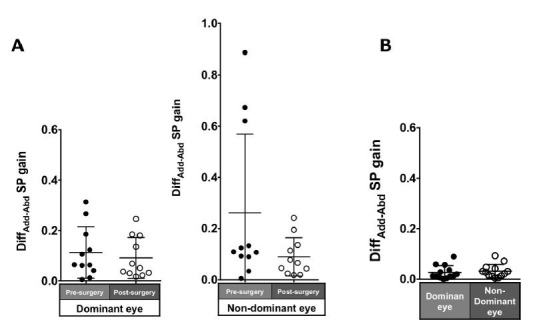
Comparison of pre- (solid black symbols) and post-surgery (open black symbols) asymmetry of adduction-abduction of both eyes in smooth pursuit gain in patients with acquired comitant esotropia (A). Asymmetry of adduction-abduction of both eyes in saccade gain in normal participants (B). Lines indicate mean with standard deviation. Diff_Add−Abd_: difference between adduction gain and abduction gain Add: adduction, Abd: abduction, SP: smooth pursuit.

Patients with ACE: The asymmetry of adduction-abduction post-surgery in the pursuit gain in each eye was smaller than pre-surgery but was not significant (dominant eye: 0.11 ± 0.1 to 0.09 ± 0.08, P = 0.7; non-dominant eye: 0.26 ± 0.31 to 0.09 ± 0.07, P = 0.21; Figure 7A).

Normal individuals: The Diff_ADD-ABD_ gain in the normal individuals was small (dominant eye: 0.07 ± 0.06; non-dominant eye: 0.09 ± 0.08; Figure 7B).

### Binocular coordination

**Figure 8.**
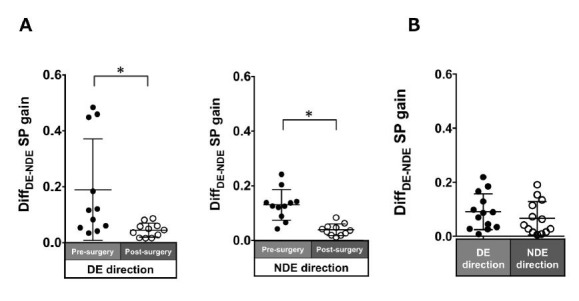
Comparison of pre- (solid black symbols) and post-surgery (open black symbols) binocular coordination of both eyes in smooth pursuit gain in patients with acquired comitant esotropia (A). Binocular coordination of both eyes in pursuit gain in normal participants (B). Lines indicate mean with standard deviation. The asterisk (*) indicates P < 0.05. Diff_DE−NDE_: difference between dominant gain and non-dominant eye gain, DE: dominant eye, NDE: non-dominant eye, SP: smooth pursuit.

Patients with ACE: In the pre-surgical data, Diff_DE-NDE_ gain in the dominant and non-dominant eye directions were 0.19 ± 0.18 and 0.13 ± 0.06, respectively. These values significantly improved post-surgery (dominant eye direction: 0.04 ± 0.03, P = 0.005; non-dominant eye direction: 0.04 ± 0.02, P = 0.003; Figure 8A).

Normal individuals: The Diff_DE-NDE_ gain was small (dominant eye direction: 0.03 ± 0.03; non-dominant eye: 0.03 ± 0.03; Figure 8B).

### Association between improvement in stereopsis and changes in eye movements after surgery

We compared five and six patients with improved and unchanged stereopsis, respectively. For the asymmetry and binocular coordination of saccades, there was no significant difference in Diff_Add-Abd_ and Diff_DE-NDE_ between the two groups after surgery, except Diff_Add-Abd_ in the dominant eye (Diff_Add-Abd_; dominant eye direction and non-dominant eye direction, P=0.02 and P=0.93, Diff_DE-NDE_; dominant eye direction and non-dominant eye direction, P=0.65 and P=0.93). For smooth pursuit, Diff_DE-NDE_ was significantly smaller in the improvement of the stereopsis group than in the unchanged group, suggesting that the improvement of binocular coordination affected the improvement of stereopsis after surgery (dominant eye direction and non-dominant eye direction, P=0.01 and P=0.02). On the other hand, Diff_Add-Abd_ was not significantly different between the two groups after surgery (dominant eye direction and non-dominant eye direction, P=1.0 and P=0.78).

## Discussion

### Saccadic eye movement

In this study, the saccade in normal individuals showed higher gain in adduction than that of abduction (adduction dominancy) but not higher asymmetry. Since the visual target appeared at a distance of 40 cm in front of the eye, it is speculated that the interaction of convergence may be related to adduction dominancy (25). In some studies, data of normal individuals without strabismus tended to show adduction dominancy (21; 12).

In this study, horizontal saccades gain in patients with ACE varied widely depending on the patient in each direction, particularly in the abduction of the dominant eye and adduction of the non-dominant eye. After surgery, the variabilities of saccade gain became small in each direction. Postoperatively, the normalization of the ocular position might resulted in a similar saccade velocity and gain in both eyes and reduced excessive preoperative gain in the dominant eye. Unlike the normal participants, in the adduction-abduction asymmetry of saccade gain, there was no tendency of adduction dominancy in patients with ACE in pre- or post-surgery (at least 3 months). However, the present study followed the patients up to 3 months postoperatively, and further long-term postoperative observation may reveal an adduction dominance.

In patients with ACE, the binocular coordination of saccades was unexpectedly poor before surgery, although it improved significantly after surgery. The binocular coordination in saccades toward the dominant eye direction was worse than that toward the non-dominant eye direction.

Based on the target's position, a saccade signal is generated and executed in the frontal eye field (16). The next fixation point is determined while generating the saccades signal since visual feedback is not perceived just before and during saccades (23; 5). In addition, the accuracy of saccades develops in infancy (1; 24). We speculated that the gain of horizontal saccades would be unaffected in patients with the onset of constant esotropia beyond the infantile period. Therefore, our results were unexpected.

These results in patients with esotropia might be caused by failure of balance of both eyes in saccades signal generation, excessive convergence, and/or the slightly tight medial rectus muscle in the esotropia eye due to longstanding deviation; however, this pathological mechanism was not explored here.

Bucci et al. reported improvement in saccade binocular coordination after strabismus surgery (3). However, they did not provide distinctions between adduction and abduction or between patients with esotropia and exotropia. Furthermore, they did not describe binocular vision function in patients with esotropia. Therefore, the results of these previous studies cannot be directly compared with the present results.

### Smooth pursuit

In this study, the smooth pursuit gain in normal individuals was similar in both dominant and non-dominant eyes and directions. There was no difference between adduction and abduction, suggesting symmetry in the gain of adduction-abduction, unlike with saccades. It is assumed that visual feedback is always applied to ascertain the moving target's position and coordinate the position of both eyes during smooth pursuit (9). Therefore, the binocular coordination of smooth pursuit was good in normal individuals.

The asymmetry of the smooth pursuit gain in patients with ACE pre-surgery was small. Sokol et al. reported that the asymmetry in patients with early-onset esotropia was greater than that in patients with late-onset esotropia (20). This asymmetry is characterized by a larger gain for nasally directed targets than for temporally directed targets (20). This may be because most patients in our study tended to fixate on the moving target using the dominant eye, while patients with early-onset esotropia, such as infantile esotropia, often show cross-fixation during smooth pursuit (7). However, major differences were viewing conditions between our study (binocular viewing) and Sokol’s study (monocular viewing) and pathological mechanisms between ACE and infantile esotropia.

In patients with ACE, the difference in pursuit gain between the left and right eyes became significantly smaller after surgery, suggesting improved binocular coordination of smooth pursuit. Similar results were obtained in a previous study on changes in pursuit gain before and after surgery in patients with intermittent exotropia (7). The correction of ocular alignment and the recovery of binocular visual function may have improved the binocular coordination of smooth pursuit (8; 11) All patients in the present study showed binocular function after surgery, suggesting that the binocular coordination of smooth pursuit is influenced by visual feedback. In particular, patients with improved stereopsis had better binocular coordination in pursuit gain after strabismus surgery.

### Limitations

This study's limitations include comparing results only up to 3 months post-surgery; long-term outcomes need to be investigated. In addition, the sample size was small (11 patients with ACE); larger sample sizes should be considered in the future. Further, only the initial saccade was analyzed, while the corrective saccades and saccade endpoints were excluded. We assumed that the steps to prevent early saccades in smooth pursuit are the same as those for ACE patients and normal subjects without strabismus. However, regarding the step-ramp method, verifying the validity of applying the steps of normal controls to ACE patients is necessary. In this study, saccade and smooth pursuit amplitude were 18.3 degrees; typically, movements greater than 15 degrees off midline also provoke head movement. Therefore, head movement might be not completely prevented, even when using a head positioner.

### Conclusion

In patients with ACE who presented after developing normal binocular vision function, the binocular coordination of saccades as well as smooth pursuit was poor. Binocular coordination of saccades and smooth pursuit seem to be improved due to the recovery in ocular deviation angle and binocular visual function after surgery leading to mechanically and functionally balanced positions of both eyes.

### Ethics and Conflict of Interest

The authors declare that the contents of the article are in agreement with the ethics described in http://biblio.unibe.ch/portale/elibrary/BOP/jemr/ethics.html and that there is no conflict of interest regarding the publication of this paper.
